# Atypical memory B cells increase in the peripheral blood of patients with breast cancer regardless of lymph node involvement

**DOI:** 10.1186/s12865-024-00620-4

**Published:** 2024-05-03

**Authors:** Atefeh Azizi, Fereshteh Mehdipour, Morteza Samadi, Reza Rasolmali, Abdol-Rasoul Talei, Abbas Ghaderi

**Affiliations:** 1 Department of Immunology, School of Medicine, Shahid Sadoughi University of Medical Sciences, Yazd, Iran; 2 Shiraz Institute for Cancer Research, School of Medicine, Shiraz University of Medical Sciences, Shiraz, Iran; 3 Department of Immunology, School of Medicine, Isfahan University of Medical Sciences, Isfahan, Iran; 4 Department of Pathology, Shiraz Central Hospital, Shiraz, Iran; 5 Breast Diseases Research Center, Shiraz University of Medical Sciences, Shiraz, Iran; 6 Department of Immunology, School of Medicine, Shiraz University of Medical Sciences, Shiraz, Iran

**Keywords:** B cells, Atypical memory B cells, Peripheral blood mononuclear cells, Breast cancer

## Abstract

**Background:**

Breast cancer is the most common cancer in females. The immune system has a crucial role in the fight against cancer. B and T cells, the two main components of the adaptive immunity, are critical players that specifically target tumor cells. However, B cells, in contrast to T cells, and their role in cancer inhibition or progression is less investigated. Accordingly, in this study, we assessed and compared the frequency of naïve and different subsets of memory B cells in the peripheral blood of patients with breast cancer and healthy women.

**Results:**

We found no significant differences in the frequencies of peripheral CD19^+^ B cells between the patients and controls. However, there was a significant decrease in the frequency of CD19^**+**^IgM^**+**^ B cells in patients compared to the control group (*P*=0.030). Moreover, the patients exhibited higher percentages of atypical memory B cells (CD19^+^CD27^‒^IgM^‒^, *P*=0.006) and a non-significant increasing trend in switched memory B cells (CD19^+^CD27^+^IgM^‒^, *P*=0.074). Further analysis revealed a higher frequency of atypical memory B cells (aMBCs) in the peripheral blood of patients without lymph node involvement as well as those with a tumor size greater than 2cm or with estrogen receptor (ER) negative/progesterone receptor (PR) negative tumors, compared with controls (*P*=0.030, *P*=0.040, *P*=0.031 and *P*=0.054, respectively).

**Conclusion:**

Atypical memory B cells (CD19^+^CD27^‒^IgM^‒^) showed a significant increase in the peripheral blood of patients with breast cancer compared to the control group. This increase seems to be associated with tumor characteristics. Nevertheless, additional research is necessary to determine the precise role of these cells during breast cancer progression

## Introduction

Cancer is a primary cause of death around the world. The most common types of cancer in 2020 were breast and lung cancers, accounting for 11.7% and 11.4% of cases, respectively [1]. Breast cancer continues to be a significant cause of cancer-related mortality, particularly among women in the developing countries [2, 3]. Therefore, international efforts and public health initiatives are primarily focused on improving prevention, screening, early detection, and treatment methods [4].

Finding biomarkers for early identification, prognosis prediction, and more effective immunotherapy and therapeutic response prediction is essential. In this way, understanding the relationship between the immune system and tumors is necessary for the development of new immunological diagnosis and therapy. Since cancer is a systemic disorder, the concentration of soluble factors and cellular components in the peripheral blood changes as the disease progresses [5]. B cells are an important subset of leukocytes that infiltrate many solid tumors. However, their role remains controversial, due to conflicting reports of both pro- and anti-tumorigenic activities [6]. Patients frequently have antibodies against antigens linked to tumors, although these antibodies do not always provide protection [7]. The microenvironment of the tumor has a lower number of infiltrating B cells compared to T cells. Nevertheless, recent studies indicate that the existence and function of B cells may be linked to the onset of carcinogenesis [8]. The presence of B cells in the tumor tissues of renal cell carcinoma, prostate and bladder cancer was associated with a poor prognosis [9–11]. On the other hand, the formation of tertiary lymphoid structure (TLS) within the tumor microenvironment (TME), which are ectopic lymphoid structures, facilitates a close interaction between T and B cells and is a positive predictive factor for the prognosis of melanoma and ovarian cancer [12, 13]. The presence of B cells among the tumor-infiltrating lymphocytes (TILs) has been linked to an improved disease prognosis in several solid tumors, including breast, colorectal, cervical, lung, and ovarian cancer [14]. Several studies have found a direct correlation between CD20^**+**^ B cells and T cells (CD4^**+**^ and especially CD8^**+**^) in the tumor tissues of various malignancies. Patients with infiltrating ductal carcinoma (IDC) had tumor samples that contained CD20^**+**^ B cells in the germinal center like structures along with CD3^**+**^ T cell zones and follicular dendritic cells (FDCs) [15]. In 2014, Garaud et al. found that B cells form TLS within breast cancer tissues and approximately 50% of these B cells exhibited a memory phenotype [16].

As mentioned before, all forms of malignancies are systemic in nature, including both leukemia and solid tumors. It is important to note that these diseases not only affect the primary site of the tumor but also have far-reaching effects on distant areas [17]. Recent studies have demonstrated that alterations in cytokine signaling, immune cell functions, differentiation, and mobilization occur in both primary and secondary lymphoid organs [18]. Several malignancies have been associated with a decreased frequency of T cells, reduced diversity of T cell receptors (TCR), and modified T cell function in the peripheral blood [19–21]. Furthermore, in breast cancer patients, peripheral CD4^+^ and CD8^+^ T cells release lower levels of IL-2 and IFN-γ in response to stimulation compared to T cells in the peripheral blood of healthy individuals [19, 20, 22]. Additionally, the presence of tumor-specific B cells, plasmablasts, plasma cells, and other B cell subtypes in the peripheral blood provides evidence for systemic B cell responses to tumor antigens [23].

B cells can be classified based on their developmental or functional state. Naïve B cells are defined as B cells that have not been exposed to antigens or gone through germinal center reaction. They do not express CD27 and exhibit IgM and IgD expression [24, 25]. Antigen stimulation promotes the formation of long-lived plasma cells and memory B cells (MBC) from the pool of naïve B cells [26, 27]. A typical characteristic of MBCs is the expression of CD21 and CD27 markers. These CD21^**+**^CD27^**+**^ B cells quickly develop into plasma cells that secrete antibodies and exhibit a high degree of affinity maturation [28, 29]. MBCs can be divided to two distinct subsets: switched memory B cells which express classes of antibodies other than IgM (CD19^**+**^CD27^**+**^IgM^**–**^) and unswitched memory B cells (CD19^+^CD27^+^IgM^+^) [30].

A subset of MBCs was identified that differs from conventional memory B cells in that they do not express CD27 and CD21 (CD21^**–**^CD27^**–**^). Due to this unique characteristic, they were named atypical memory B cells (aMBC) [31]. aMBCs were first identified as tissue-like memory B cells in tonsil tissues [32]. There have also been reports of B cells with the CD21^low^CD27^**–**^FCRL4^**+**^ phenotype in the peripheral blood of human with immunodeficiency virus (HIV) infection [33, 34]. In conditions like HIV infection, hepatitis C [35], malaria [36], autoimmune disorders [37], and cancer [38–40] that cause long-term antigen stimulation, large frequencies of aMBCs are seen. Approximately, 3-5% of peripheral blood B cells are made up of aMBCs in healthy individuals, but in chronic conditions, this percentage can rise up to 50% of all circulating B cells [33]. In previous investigations, it has been shown that these cells have a lower capacity to proliferate and produce antibodies and cytokines when stimulated through the BCR, compared to the CD21^**+**^CD27^**+**^ conventional memory B cells. They were named exhausted B cells because they became anergic due to repeated stimulation by antigens [33]. In individuals with non-small cell lung cancer, there is a negative association between CD21^**–**^ CD27^**–**^ B cells and the effectiveness of immune checkpoint blockade therapy. This finding suggests that these B cells share similar characteristics to exhausted B cells seen in chronic infections [39]. In contrast, in patients with hepatocellular carcinoma and high grade serous ovarian tumors, tumor-infiltrating B lymphocytes exhibited an atypical memory phenotype (CD27^**–**^IgD^**–**^). Their presence was associated with increased survival and reduced rate of recurrence [38, 40].

In general, aMBCs represent a heterogeneous population both within individuals and across different diseases [33]. Since these studies have been conducted under different conditions, there is no consensus regarding the exact nature and function of aMBCs. Additionally, B cells and their subtypes in the peripheral blood of patients with cancer have received less attention than myeloid and T cells. Therefore, in this study, we examined the peripheral blood of patients with breast cancer and healthy individuals to investigate the possible differences in their conventional and atypical memory B cells.

## Results

### Frequency of CD19^+^ B and B cell subsets in the peripheral blood of patients with breast cancer and controls

**Fig. 1:**
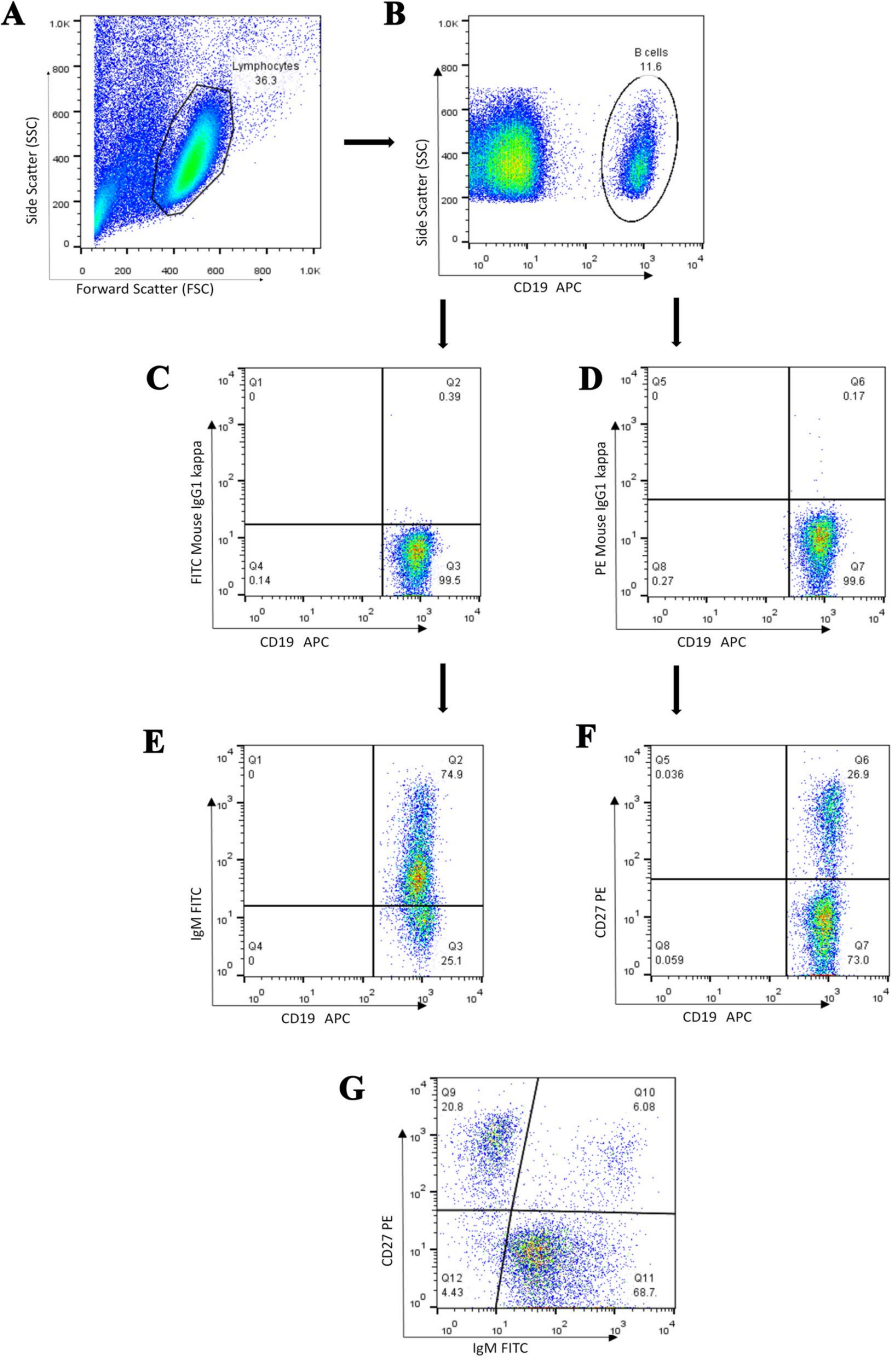
Representative flow cytometry analysis of B cell subpopulations in the peripheral blood of patients with breast cancer. **A.** Lymphocytes were gated based on their forward and side scatters, and **B.** the percentage of CD19^**+**^ cells in the lymphocyte gate was determined. Using fluorescence minus one (FMO, graphs **C&D.**), **E**, CD19^**+**^CD27^**+**^ B cells, **F.** CD19^+^IgM^+^ B cells and **G.** various B cell subsets including naïve (CD19^+^CD27^‒^IgM^+^), unswitched memory (CD19^+^CD27^+^IgM^+^), switched memory (CD19^+^CD27^+^IgM^‒^) and atypical memory (CD19^+^CD27^‒^IgM^‒^) B cells were evaluated within the CD19^**+**^ gate

**Fig. 2:**
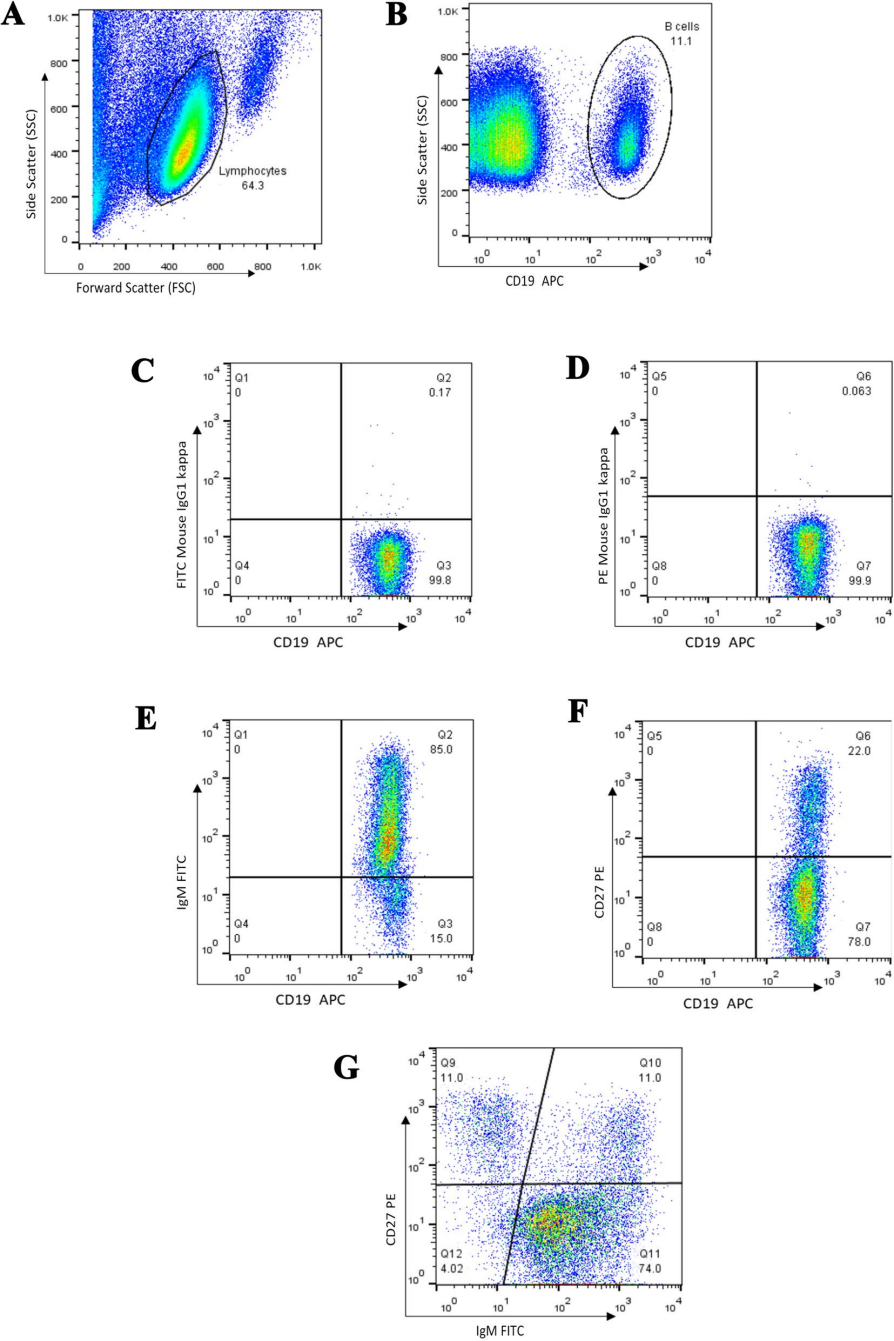
Representative flow cytometry analysis of B cell subpopulations in the peripheral blood of healthy individuals. **A.** Lymphocytes were gated based on their forward and side scatters, and **B.** the percentage of CD19^**+**^ cells in the lymphocyte gate was determined. Using fluorescence minus one (FMO, graphs **C&D.**), **E**, CD19^**+**^CD27^**+**^ B cells, **F.** CD19^+^IgM^+^ B cells and **G.** various B cell subsets including naïve (CD19^+^CD27^‒^IgM^+^), unswitched memory (CD19^+^CD27^+^IgM^+^), switched memory (CD19^+^CD27^+^IgM^‒^) and atypical memory (CD19^+^CD27^‒^IgM^‒^) B cells were evaluated within the CD19^**+**^ gate

**Fig. 3:**
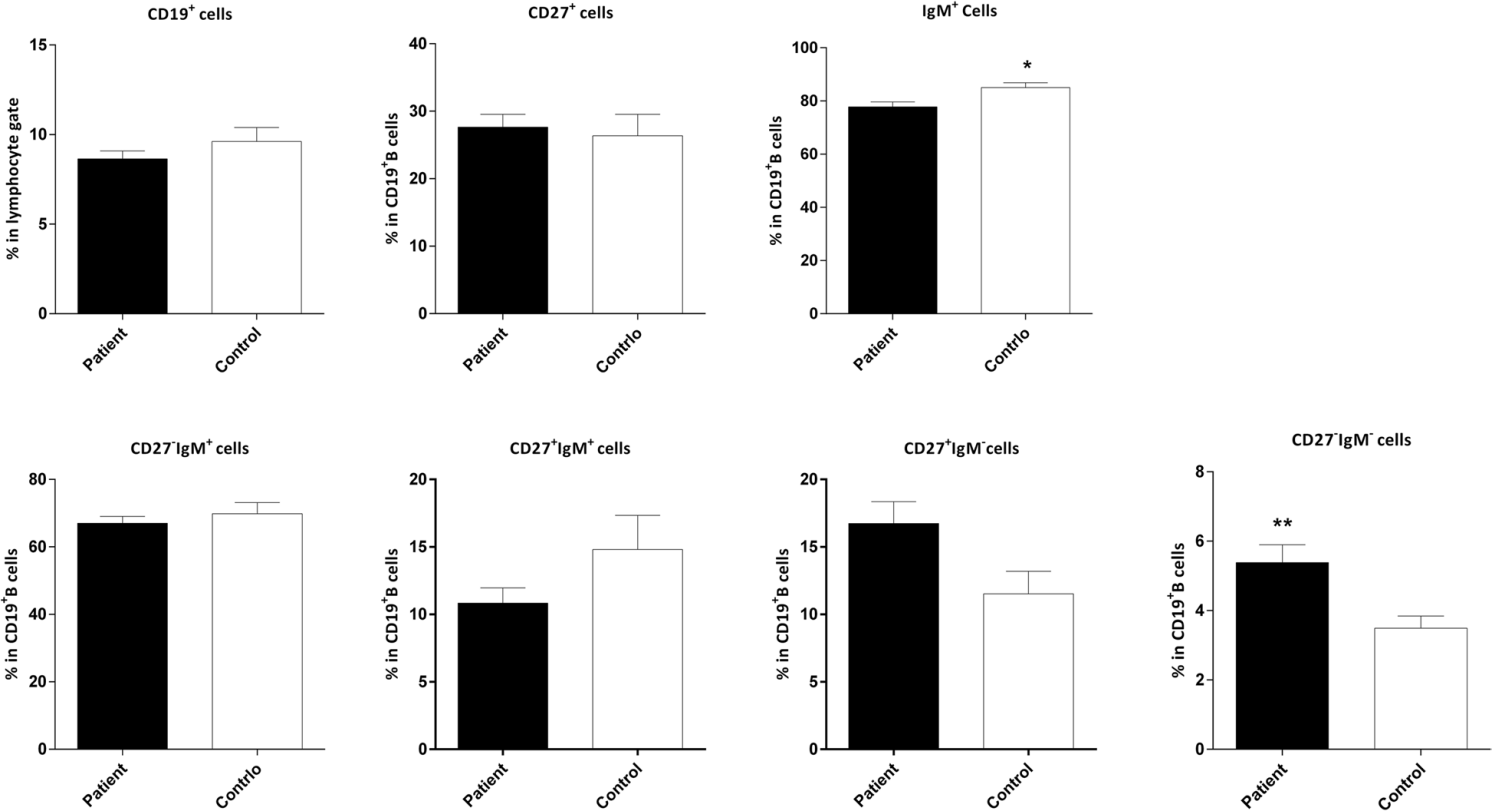
Comparison of the frequencies of CD19^+^ B and B cell subsets in the peripheral blood of patients with breast cancer and controls. The data is shown as Mean ± SEM. *P value < 0.05, ** *P* value < 0.01. SEM: standard error of the mean

We used CD19 as a pan B cell marker and compared the frequency of CD19^**+**^ B cells in the lymphocyte gate in 52 patients and 25 age-matched contols (Tables 1 and 2). The results showed no significant difference between the two groups. In the next step, we assessed naïve (CD19^+^CD27^‒^IgM^+^), unswitched memory (CD19^+^CD27^+^IgM^+^), switched memory (CD19^+^CD27^+^IgM^‒^), and atypical memory (CD19^+^CD27^‒^IgM^‒^) B cells in the CD19^**+**^ gate and compared them in the two groups (Fig. 1and 2, Table 3). The frequency of CD19^**+**^IgM^**+**^ B cells was lower in the patients compared to the control group (*P*=0.030). Additionally, the percentages of switched memory B cells (CD19^+^CD27^+^IgM^‒^) and atypical memory B cells (CD19^+^CD27^‒^IgM^‒^) were higher in the patients, however the P value for the former did not reach the statistical significance (*P*=0.074 and *P*=0.006, respectively, Fig. 3).

**Table 1:** The information of study groups

Study group	Age (years)	
	Median (min-max)	Mean± SD
Patients (*n*=52)	54 (31-76)	53.7±12.9
Control (*n*=25)	48 (22-70)	50±13.4

**Table 2:** Clinico-pathological characteristics of breast cancer patients

**Characteristics**	**Value**
**Age (years)**	53.7± 12.9 (31-76)
**Lymph Node (LN) Status**	
N0 (Free LNs)	22 (42.3%)
N1 ( 1-3 involved LNs)	22 (42.3%)
N2 (4-9 involved LNs)	4 (7.7%)
N3 (>9 involved LNs)	2 (3.8%)
Nx (Unknown)	2 (3.8%)
**Tumor Size (greatest dimension, cm)**	
T1 (≤2)	31 (59.6%)
T2 (2-5)	16 (30.8%)
T3	1 (1.9%)
Tx (Unknown)	4 (7.7%)
**Stage**	
I	13 (25.0%)
II	26 (50.0%)
III	7 (13.5%)
Unknown	6 (11.5%)
**Histological Grade**	
Well differentiated (I)	6 (11.5%)
Moderately differentiated (II)	33 (63.5%)
Poorly differentiated (III)	9 (17.3%)
Unknown	4 (7.7%)
**Tumor Type**	
Infiltrating ductal carcinoma (IDC)	42 (80.8%)
IDC with medullary features (IDC+M)	4 (7.7%)
Others (Lobular carcinoma, Metaplastic Carcinoma)	2 (3.8%)
Unknown	4 (7.7%)
**HER2 Expression**	
Positive	10 (19.2%)
Negative	26 (50.0%)
Equivocal	7 (13.5%)
Unknown	9 (17.3%)
**ER Expression**	
Positive	31 (59.6%)
Negative	13 (25.0%)
Unknown	8 (15.4%)
**PR Expression**	
Positive	27 (51.9%)
Negative	16 (30.8%)
Unknown	9 (17.3%)

*LN* Lymph Node, *ER* Estrogen Receptor, *PR* Progesterone Receptor, *HER2* Human Epidermal Growth Factor Receptor 2

**Table 3:** The percentages of B cells and B cell subsets in the peripheral blood of patients and controls

Cell subsets	**Min**		**Max**		**Median**		**Mean±SD**	
	C	Pt	C	Pt	C	Pt	C	Pt
CD19^**+**^ cells (in lymphocytes’ gate)	3.5	2.5	19.5	17.0	9.2	7.7	9.6±3.9	8.6±3.1
CD19^**+**^CD27^**+**^ cells (in B cells’ gate)	5.7	4.7	60.8	57.6	25.5	24.6	26.4± 15.8	27.6±13.6
CD19^**+**^IgM^**+**^ cells (in B cells’ gate)	61.1	47.9	98.3	96.1	84.9	80.3	85±9.1	77.8±13.4
CD19^+^CD27^‒^IgM^+^ cells (in B cells’ gate)	35.2	35.6	93.6	91.2	70.8	71.1	69.8± 16.6	67.0±14.7
CD19^+^CD27^+^IgM^+^ cells (in B cells’ gate)	1.8	0.9	47.9	39.1	9.0	9.3	14.8± 12.7	10.8±8.2
CD19+CD27+IgM^‒^ (in B cells’ gate)	0.7	1.2	34.1	46.8	10.2	14.9	11.5± 8.4	16.7±11.6
CD19^+^CD27^‒^IgM^‒^ (in B cells’ gate)	0.7	1.9	8.0	24.8	3.1	4.6	3.5±1.7	5.4±3.7

*C* Control, *Pt* Patients

Analyses revealed no significant differences in the frequency of naïve (CD19^+^CD27^‒^IgM^+^) and unswitched memory B cells (CD19^+^CD27^+^IgM^+^) between patients and controls.

### Frequency of CD19+ B and B cell subsets in the peripheral blood of controls and patients in different stages and with different nodal status

**Fig. 4:**
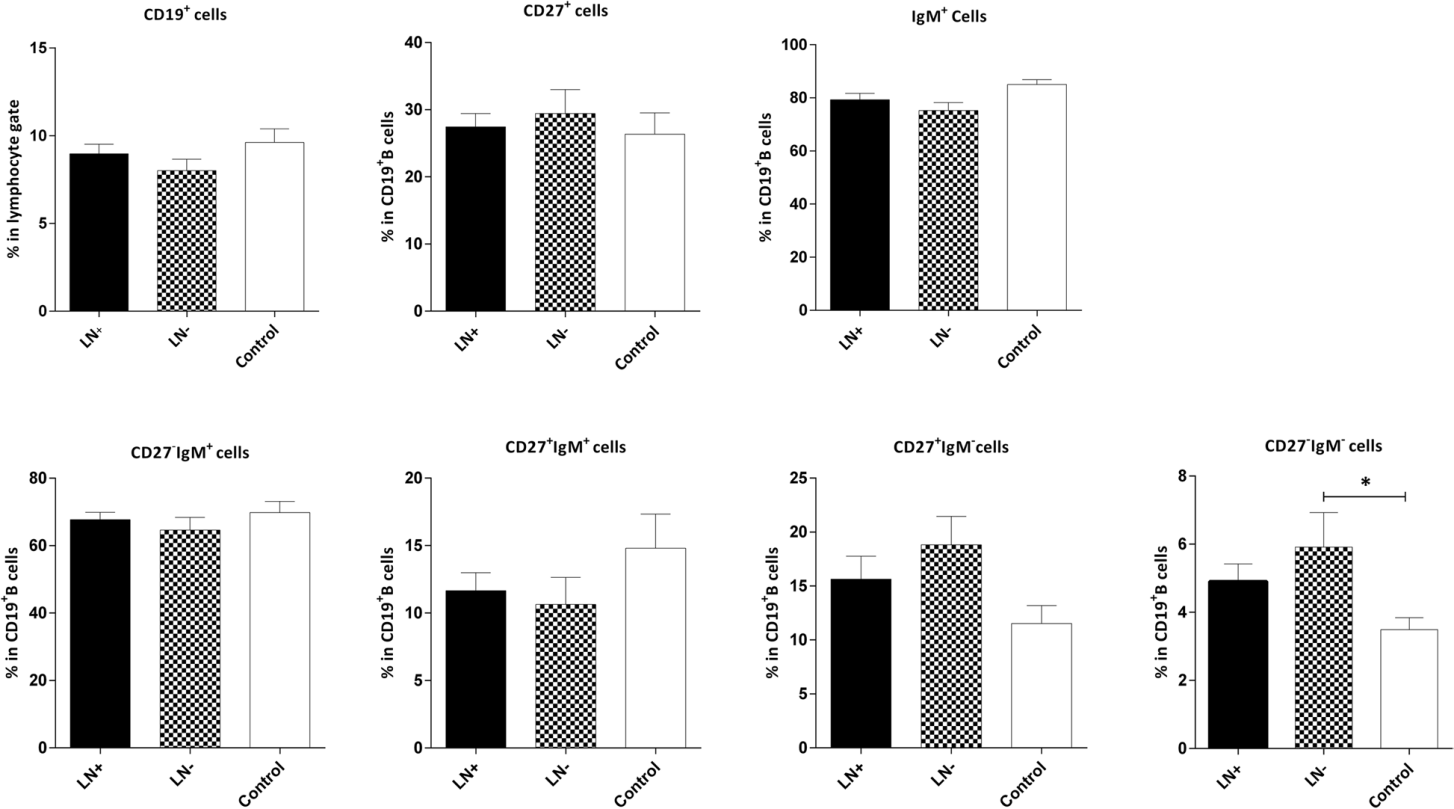
Comparison of the frequencies of CD19^+^ B and B cell subsets in the peripheral blood of controls and patients with and without lymph node (LN) involvement. LN‒ (patients without LN involvement) and LN+ (patients with at least one involved LN). The data is presented as Mean ± SEM. *P value<0.05. SEM: standard error of the mean

The frequencies of B cells and their subsets showed no significant differences between patients in different stages and the control group. Furthermore, our analysis revealed no significant difference in the frequencies of B cells and their naïve or memory subsets between LN‒ (without LN involvement) and LN+ (with at least one involved LN) patients. However, the percentage of CD19^**+**^IgM^**+**^ B cells was slightly lower in the LN‒ patients compared to the control group, although the difference was not statistically significant (P=0.051). Furthermore, the frequency of aMBCs (CD19^+^CD27^‒^IgM^‒^) was higher in both LN‒ and LN+ patients, but the P value was only significant for the former group (*P*=0.030, Fig. 4).

### Frequency of CD19+ B and B cell subsets in the peripheral blood of controls and patients based on tumor size (T), grade, and disease stage

**Fig. 5:**
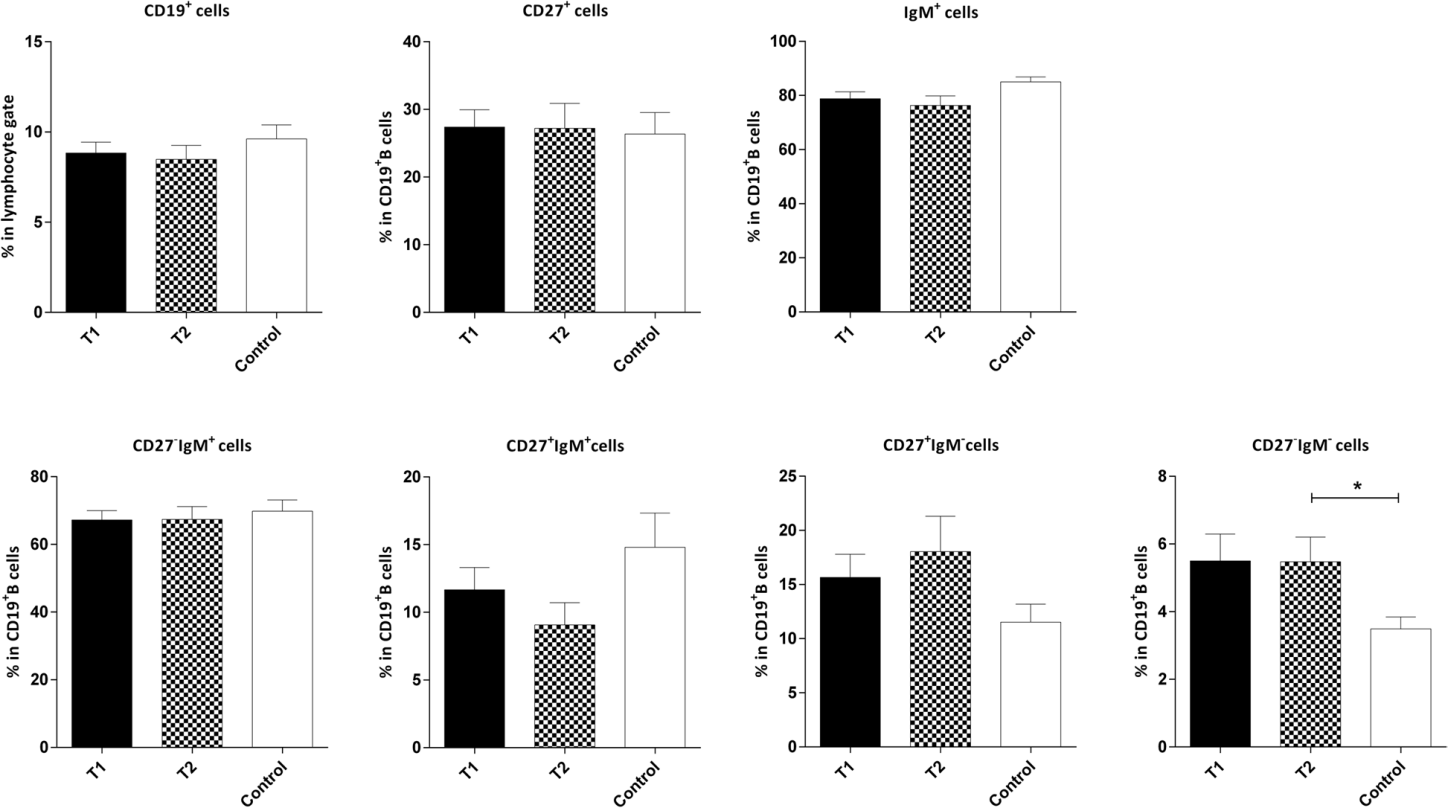
Comparison of the frequencies of CD19^**+**^ B and B cell subsets in the peripheral blood of controls and patients with breast cancer based on the tumor size. Tumor size was categorized as ≤ 2cm (T1) and > 2cm (T2). Data are shown as Mean ± SEM. *P value< 0.05. SEM: standard error of the mean

The frequencies of aMBCs (CD19^+^CD27^‒^IgM^‒^) were higher in patients with tumor size ≤2cm (T1) and tumor size >2cm (T2) in comparison to the control group. However, it is noteworthy that the difference was only significant in the T2 group (*P*=0.070 and *P*=0.040 for T1 and T2, respectively), as shown in Fig. 5.

**Fig. 6:**
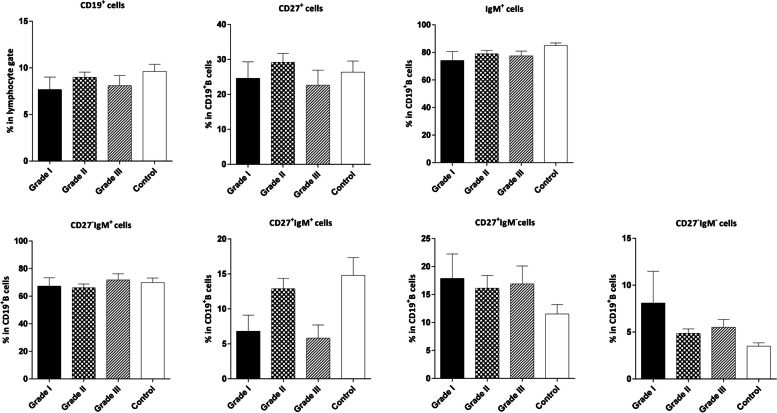
Comparison of the frequencies of CD19^**+**^ B and B cell subsets in the peripheral blood of controls and patients with varying breast tumor grades. The data is displayed as Mean ± SEM. SEM: standard error of the mean

**Fig. 7:**
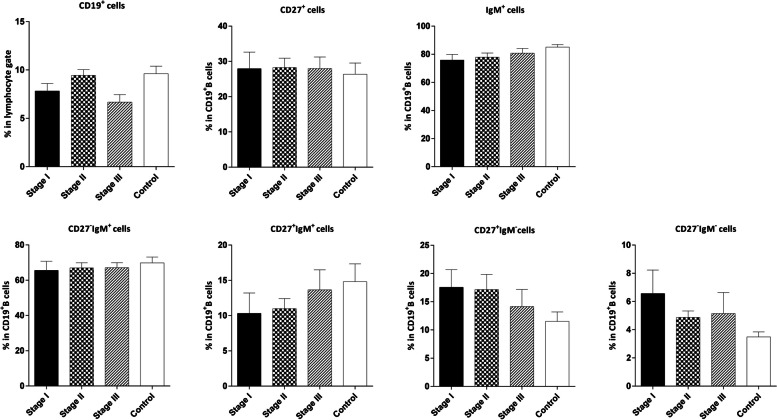
Comparison of the frequencies of CD19^**+**^ B and B cell subsets in the peripheral blood of controls and patients with different stages of breast cancer. The data is presented as Mean ± SEM. SEM: standard error of the mean

We observed no significant differences between B cells and its subsets in the patients with different breast tumor grades and at various disease stages compared to the control group (Fig. 6 and 7 respectively).

### Frequency of CD19^+^ B and B cell subsets in the peripheral blood of controls and patients with different estrogen receptor (ER), progesterone receptor (PR) and human epidermal growth factor receptor-2 (HER2) status

**Fig. 8:**
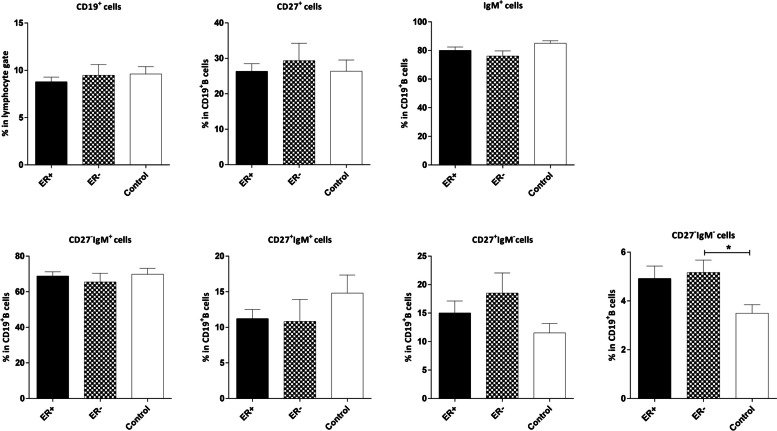
Comparison of the frequencies of CD19^**+**^ B and B cell subsets in the peripheral blood of patients with ER+/‒ breast cancer and age-matched controls. The data is presented as Mean ± SEM. *P value< 0.05. SEM: standard error of the mean. ER: estrogen receptor

**Fig. 9:**
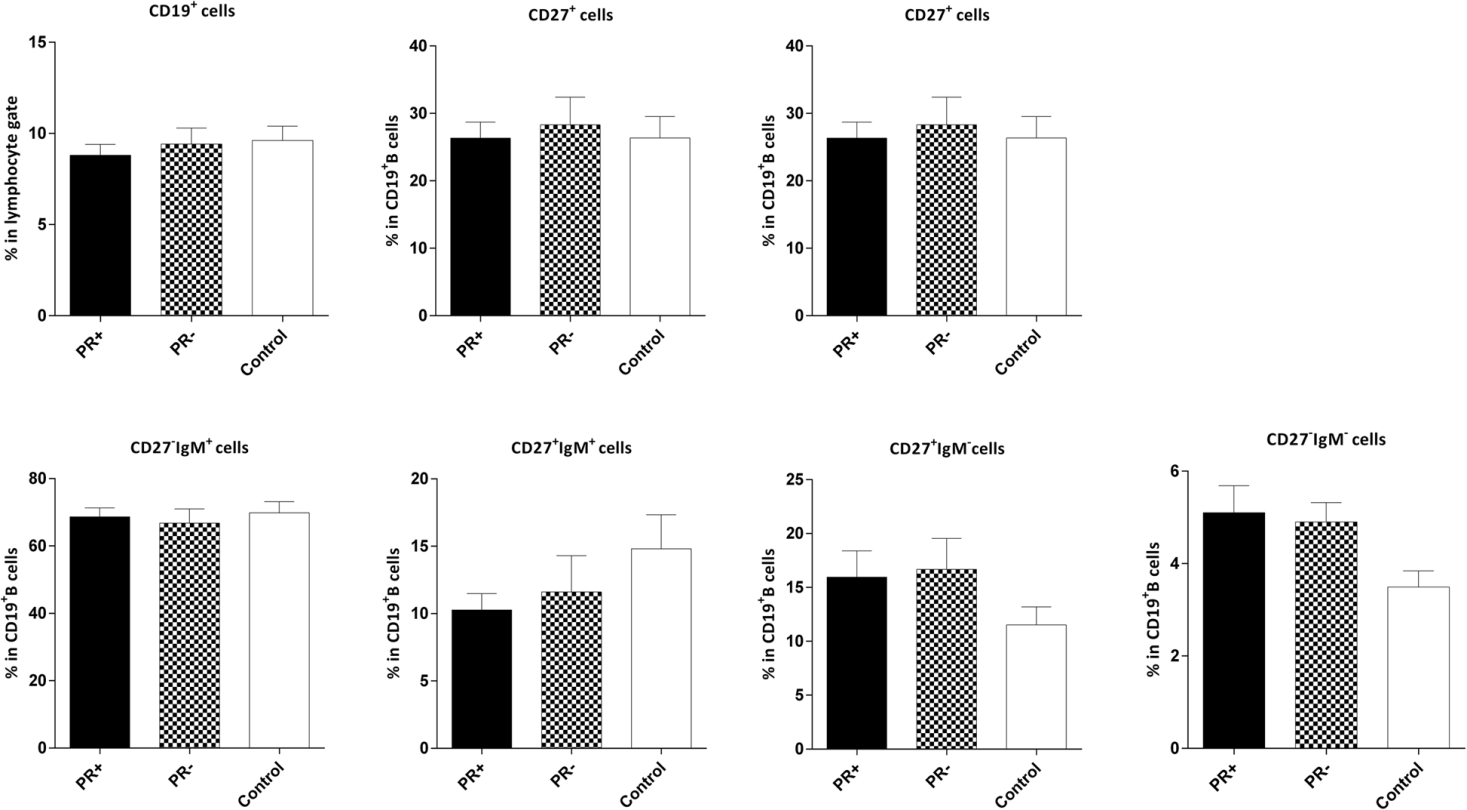
Comparison of the frequencies of CD19^**+**^ B and B cell subsets in the peripheral blood of patients with PR+/‒ breast cancer and age-matched controls. The data is demonstrated as Mean ± SEM. SEM: standard error of the mean. PR: progesterone receptor

**Fig. 10:**
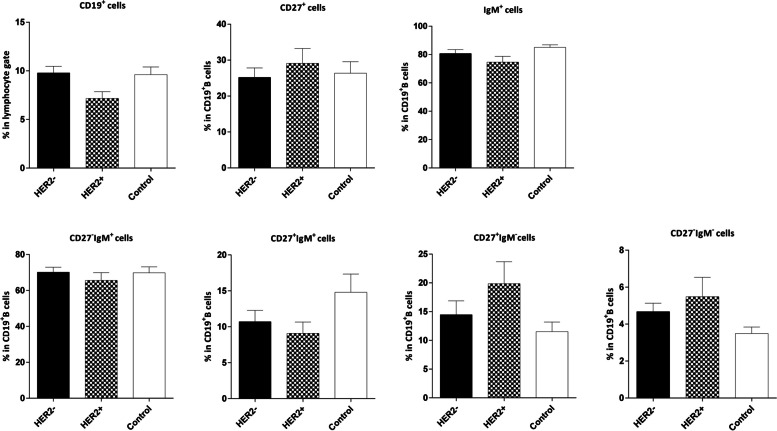
Comparison of the frequencies of CD19^**+**^ B and B cell subsets in the peripheral blood of controls and patients with HER2+/– breast tumor. The data is presented as Mean ± SEM. HER2: human epidermal growth factor receptor 2, SEM: standard error of the mean

The analysis revealed no significant association between the frequencies of B cells and their naïve/memory subpopulations and the expression of ER/PR and HER2 in the tumor cells. However, the frequency of aMBCs (CD19^+^CD27^‒^IgM^‒^) was higher in both ER– and PR– patients compared to the control group, although the difference was not statistically significant in the PR–patients (*P*=0.031 and *P*=0.054, respectively; Figs 8, 9 and 10).

## Discussion

As previously indicated, breast cancer continues to be the most common cancer among women worldwide. Despite all efforts, therapeutic approaches are not completely effective, and the mortality rate remains high [41]. The presence of tumors and immune cells that respond to tumors, as well as their mediators, such as cytokines can be observed in the peripheral blood during tumor development and progression [18, 42–45]. This suggests that these markers can be utilized for early diagnosis, evaluating patients’ response to treatment, and predicting overall survival (OS) [46]. While most studies have focused on the relationships between the innate immune and T cells and cancer, B cells have largely been ignored. This may be due to the long-held belief that B cells solely function as antibody producers, leading to their diverse functions being either unknown or overlooked [47, 48].

B lymphocytes are typically concentrated at the periphery of tumors and frequently found in the lymph nodes proximal to the tumor microenvironment [49]. The presence of the tumor-specific B cells, such as plasmablasts and plasma cells, in the peripheral blood indicates a systemic response of B cells to tumor antigens [23]. In this study, we examined the frequencies of B cells and their naïve, switched, unswitched, and atypical memory populations in patients with different stages of breast cancer and compared them with healthy age/sex-matched individuals. Our goal was to understand the changes in B cell subsets in the peripheral blood, during the development and progression of breast cancer.

Our results showed no significant difference in the frequency of CD19^**+**^ B cells in the peripheral blood of patients with breast cancer compared to the control group. Similarly, there was no significant change in the percentage of B cells among patients in different disease stages or with various tumor grade, tumor size, or nodal status.

In contrast to reports of lower frequency of peripheral CD4^**+**^ T cells in breast cancer patients, almost all studies have found no significant difference in the frequency of peripheral CD19^**+**^ B cells between cancer patients and healthy controls. For example, Zirakzadeh et al. have shown that the frequency of peripheral blood B cells did not change in patients with various solid tumors, such as colon cancer, malignant melanoma, pancreatic cancer and prostate cancer compared to healthy individuals [50]. According to another study the frequency of CD19^**+**^ B cells in the peripheral blood of breast cancer patients was not significantly different from that of age-matched healthy controls before chemotherapy. However, the administration of chemotherapy resulted in a reduction in the absolute count of peripheral B cells in patients compared to their initial pre-treatment condition [51]. In contrast to these investigations, one study found that patients with breast cancer had an increased frequency of peripheral CD19^**+**^ B cells [52]. It is important to note that their sample size was smaller than ours (27 patients and 12 controls).

Another finding of this study was that CD19^**+**^IgM^**+**^ B cells which includes naïve (CD19^+^CD27^‒^IgM^+^) and unswitched memory B cells (CD19^+^CD27^+^IgM^+^), decreased in patients compared to the control group. However, further analysis revealed that there was no difference in the frequency of unswitched memory B cells (CD19^+^CD27^+^IgM^+^) between patients and controls. Contrarily, an increasing trend was observed in switched memory B cells (CD19^+^CD27^+^IgM^‒^) among the patients compared to the control group. This result was somehow expected because cancer causes general inflammation which leads to B cell activation and class switching. Interestingly, even in the advanced stages and after LN involvement, B cell class switching is not disturbed as the frequency of switched memory B cells was not different in the peripheral blood of patients compared to controls. However, our recent study, which evaluated the frequencies of memory and class switched B cells in the breast tumor-draining lymph nodes (TDLNs), revealed that the frequencies of these subsets were significantly lower in the involved lymph nodes compared to the uninvolved ones [53]. This apparent discrepancy stems from the lack of examination of normal LNs due to the ethical and legal considerations in the study of the TDLNs. However, it is important to bear in mind that a non-metastatic LN is a reactive environment that responds to tumor antigens and is more active than a normal LN. As a result, it contains a higher number of memory and class switched B cells. In contrast, in a metastatic LN, the immune system is suppressed and the frequency of active/memory and class switched B cells decreases to the level of a normal nonreactive LN. This observation is further supported by the fact that the frequency of unswitched B cells decreased significantly in the LN‒ patients but not in the LN+ patients compared to the control group.

In this study, the frequency of CD19^+^CD27^‒^IgM^‒^ aMBCs was significantly higher in the peripheral blood of patients with breast cancer than in the control group. Notably, the percentage of these cells was higher in both LN‒ and LN+ patients compared to the control group, suggesting that the frequency of aMBCs increases in patients even before lymph node involvement. Moreover, the frequency of these cells was higher in patients with tumors larger than 2cm (T2) and those with ER‒/PR‒ tumors compared to the healthy controls. Therefore, there might be a link between the increase of aMBCs and tumor characteristics, such as size or hormone receptor expression. However, this hypothesis needs further investigation.

In contrast to studies on viral and parasitic infections, a higher frequency of CD27^**‒**^ aMBCs along with CD8^**+**^ T cells was associated with better survival and good prognostic markers in tumor tissues of patients with ovarian and hepatocellular carcinoma [38, 40] as well as in the TDLNs of patients with head and neck squamous cell carcinoma (HNSCC) [53]. However, in the present study, the frequency of CD27^**‒**^ memory B cells did not show an association with prognostic markers. Further studies are needed to uncover the role of these cells in immunity against breast cancer.

## Conclusion

In this study, we investigated the frequencies of naïve, switched, unswitched and atypical memory B cells in the peripheral blood of patients with breast cancer and healthy controls. We found no significant difference in the frequency of circulating CD19^**+**^ B cells between patients with breast cancer and controls. Different groups of memory B cells exhibited varying frequencies in patients compared to controls. Although there was no difference in unswitched memory B cells between patients and controls, the percentages of switched memory and atypical memory B cells were higher in the patients. It is noteworthy that the frequencies of atypical memory B cells in patients increased regardless of lymph node involvement. And this difference was associated with the tumor characteristics as the frequencies of atypical memory B cells were higher in patients with larger or ER–/PR– tumors compared to controls. Furthermore, the frequency of B cell subsets changed during tumor development in the peripheral blood of patients with breast cancer, supporting the idea of a systemic immune change during cancer. However, further investigation is needed to elucidate the role of various B cell subsets in breast cancer and their potential association with prognosis.

## Materials and methods

### Subjects

We collected blood samples from 52 women with breast cancer and 25 age-matched controls (Tables 1 and 2). It should be noted that the patients did not undergo any prior treatment with chemo- or radiation therapy. The control group had no history of cancer, autoimmune diseases, or chronic infections. Furthermore, both the patients and controls showed no sign of any infectious diseases within a month before sampling, and were not taking any medications that could affect their immune profiles. Breast cancer was confirmed in all patients and we obtained clinicopathological data from the pathological reports. Additionally, the hormone receptor status of the tumors (ER, PR, and HER2) was determined through immunohistochemistry (IHC) in the pathology department of the Shiraz Central Hospital and Raz Pathobiology Laboratory and reported by expert pathologists.

It is important to emphasize that written consent was obtained from all participants, and the study protocol was approved by the Ethics Committee of Shahid Sadoughi and Shiraz Universities of Medical Sciences (IR.SSU.MEDICINE.REC.1402.014).

### Isolation and staining of the mononuclear cells

A total of 3 mL of peripheral blood was collected from both individuals with breast cancer prior to surgery and healthy controls. Mononuclear cells were obtained by centrifuging heparinized blood over Ficoll-Hypaque (Histiosep, Iran). These cells were then resuspended in a complete culture medium (RPMI-1640 containing 10% FBS and 1% Penicillin/ Streptomycin, all from Gibco, Life Technologies, USA) and added to flasks for overnight incubation. The next day, 1×10^6^ cells/ml were used to evaluate CD27 and IgM expression on B cells. The cells were washed with staining buffer (PBS+ 10% FBS) and added to a test tube along with APC-conjugated anti-CD19 antibody (Clone:HIB19, Biolegend, USA), FITC-conjugated anti-IgM antibody (Clone: MHM-88, Biolegend, USA), and PE-conjugated anti-CD27 antibody (Clone: 17A12, BD Bioscience, USA). In addition, an isotype tube was prepared by adding APC-conjugated anti-CD19 antibody, PE Mouse IgG1, kappa isotype Ctrl (Clone: MOPC-21, Biolegend, USA) and FITC Mouse IgG1, kappa isotype Ctrl (Clone: MOPC-21, Biolegend, USA). After incubating at 4°C and undergoing two rounds of washing, the cells were ready for flow cytometry acquisition.

### Flow cytometry data acquisition and analysis

We used a four-color FACSCalibur flow cytometer (BD Biosciences, USA) to collect the data. To ensure accuracy, we acquired at least 200000 cells in the test tube. The data were analyzed using Flow Jo software (Version 10.1, Ashland, OR, USA). Lymphocytes were gated based on their forward and side scatters. We used CD19, as a pan B cell marker, to determine B lymphocytes. Finally, we assessed the frequencies of B cells expressing IgM and/or CD27.

### Statistical analysis

To compare the frequencies of different cell subsets in two or more groups, we used the non-parametric Mann-Whitney U and Kruskal-Wallis H tests. For pairwise comparisons, we used Dunn’s posttest. To assess the relationship between cell subsets and tumor size or the number of involved LNs, we used Spearman's ranks correlation test. We performed the statistical analysis using SPSS 16 Software from SPSS GmbH (Germany), considering P values less than 0.05 as statistically significant. We used GraphPad Prism 6 software (Inc: San Diego CA, USA) to create the graphs.

## Data Availability

The data used to support the findings of this study are included within the article.

## Abbreviations

aMBCs: atypical memory B cells
BCR: B cell receptor
ER: Estrogen receptor
FBS: Fetal bovine serum
FDC: Follicular dendritic cell
FMO: Fluorescence minus one
HER-2: Human epidermal growth factor receptor 2
HIV: Human immunodeficiency virus
HNSCC: Head and neck squamous cell carcinoma
ICB: Immune checkpoint blockade
IDC: Infiltrating ductal carcinoma
IFN: Interferon
IHC: Immunohistochemistry
IL: Interleukin
LN: Lymph node
MBC: Memory B cell
OS: Overall survival
PBS: Phosphate buffer saline
PR: Progesterone receptor
TCR: T cell receptor
TDLNs: Tumor draining lymph nodes
TILs: tumor-infiltrating lymphocytes
TLS: Tertiary lymphoid structure
TME: Tumor microenvironment
